# Translocation of Charged Polymers through a Nanopore in Monovalent and Divalent Salt Solutions: A Scaling Study Exploring over the Entire Driving Force Regimes

**DOI:** 10.3390/polym10111229

**Published:** 2018-11-06

**Authors:** Pai-Yi Hsiao

**Affiliations:** 1 Department of Engineering and System Science, National Tsing Hua University, Hsinchu 30013, Taiwan; pyhsiao@ess.nthu.edu.tw or pyhsiao@mx.nthu.edu.tw; Tel.: +886-3-516-2247; 2 Institute of Nuclear Engineering and Science, National Tsing Hua University, Hsinchu 30013, Taiwan

**Keywords:** polyelectrolyte, translocation, scaling theory, tension propagation, drift-diffusion, molecular dynamics simulations

## Abstract

Langevin dynamics simulations are performed to study polyelectrolytes driven through a nanopore in monovalent and divalent salt solutions. The driving electric field *E* is applied inside the pore, and the strength is varied to cover the four characteristic force regimes depicted by a rederived scaling theory, namely the unbiased (UB) regime, the weakly-driven (WD) regime, the strongly-driven trumpet (SD(T)) regime and the strongly-driven isoflux (SD(I)) regime. By changing the chain length *N*, the mean translocation time is studied under the scaling form $\langle \tau \rangle ∼N^{\alpha }E^{-\delta }$. The exponents α and δ are calculated in each force regime for the two studied salt cases. Both of them are found to vary with *E* and *N* and, hence, are not universal in the parameter’s space. We further investigate the diffusion behavior of translocation. The subdiffusion exponent $\gamma_{p}$ is extracted. The three essential exponents $\nu_{s}$, *q*, $z_{p}$ are then obtained from the simulations. Together with $\gamma_{p}$, the validness of the scaling theory is verified. Through a comparison with experiments, the location of a usual experimental condition on the scaling plot is pinpointed.

## 1. Introduction

Translocation, which consists of transporting biomolecules across a membrane through small pores of nanometer size, is a fundamental and important mechanism taking place in many biological processes, such as in gene expression and viral infection [1]. Since the demonstration of the power of using membrane channels as individual polynucleotides detectors in the 1990s [2,3], the study of the translocation of bio-polymers has become a very active research domain, for the purpose of ameliorating the efficiency and costs of genome sequencing [4,5,6]. It has attracted much attention of the community because translocation involves a variety of interesting physical aspects, and the mechanism has not yet been fully understood [7,8,9]. A central question to be studied is the mean translocation time 〈τ〉, which is generally expressed in the scaling form, $\langle \tau \rangle ∼N^{\alpha }f^{-\delta }$, where *N* is the length of the polymer and *f* is the applied force to drive the translocation. Theoretical understanding of translocation started from the seminal work of Sung and Park [10]. They treated the problem with a Fokker–Planck equation and predicted α=2+ν for unbiased translocation; as a bias (due to a chemical potential difference) takes effect, the exponent transited to be 1+ν, where the exponent ν was argued to be 1 in the Rouse dynamics and 0.5 in the Zimm one. Later, several research groups dealt with the problem, focusing on different physical aspects, and obtained the exponents that were not always consistent with each other. For example, Muthukumar speculated that the diffusion coefficient of a chain through a pore should be independent of the chain length, and (α,δ) were predicted as (2,0) and (1,1) for unbiased and biased translocation, respectively [11]. Kantor and Kardar argued that the quasi-equilibrium condition should not be well held in the process and derived a lower bound for the pore-blockade time for forced translocation as $\langle \tau \rangle ∼N^{1+\nu }f^{-1}$ with ν being the Flory exponent [12]. The non-equilibrium feature of translocation has been studied by using fractional diffusion equations [13,14]. The exponent α was found to be $2+2\nu +\gamma_{1}$ under unbiased conditions where $\gamma_{1}$ is the exponent of surface entropy [14].

An important progress has been made by attributing the anomalous dynamics in unbiased translocation as a result of the imbalanced monomer densities across the two sides of the pore, giving rise to an effect called the “memory effect” [15,16]. The mean square displacement $\langle \Delta s^{2}\left(t\right)\rangle$ of the reaction coordinate s(t) was argued to increase with time *t* as $t^{\left(1+\nu )/(1+2\nu \right)}$ in the subdiffusion stage; the translocation time was then determined by studying the diffusion stage thereafter and shown to be $N^{2+\nu }$ [8,15]. The view point was later put on the imbalanced tensile force preexisting on the chain by Sakaue and coworkers [17,18,19,20]; new physical pictures were proposed, using the idea of tension propagation, to explain biased translocation. In their theory, the cis-side chain was subdivided into a moving and a quiescence domain, demarcated by the tensile front of the propagation. Depending on the strength of the driving force, the moving sub-chain exhibits different conformations, categorized as the equilibrium, the trumpet, the stem-flower and the strongly-stretching conformation. The exponent α in the four regimes was claimed to be 1+ν, $\left(1+\nu +2\nu^{2}\right)/\left(1+\nu \right)$, $\left(1+\nu +2\nu^{2}\right)/\left(1+\nu \right)$ and 1+ν, respectively, while δ was 1, 2/(1+ν), 2ν/(1+ν) and 1 [20]. Therefore, the scaling exponents of polymer translocation depend on *f* and *N* and are not universal over the parameter’s spaces. Rowghanian and Grosberg modified Sakaue et al.’s pictures and proposed an isoflux trumpet model, which gave (α,δ)=(1+ν,1) for biased translocation and (1+2ν,0) for the unbiased one in the Rouse dynamics [21]. Dubbeldam et al. classified the motions of the moving sub-chain into the trumpet, the stem-trumpet and the stem regimes [22,23]. They found that α transited from 2ν to 1+ν either by increasing the driving force or by growing the chain length in the three regimes. However, the value of δ was always one. The asymmetrical dynamics, owing to the difference in pulling and pushing the chain, respectively, at the pore entrance and the pore exit, has been noticed and treated [23,24]. The translocation was investigated by using a similar model to take into account the crowding effect of the monomers leaving the pore. Recently, Sakaue refined his theory and successfully formulated a regime, called the “weakly-driven regime”, which bridges the gap of the usual theoretical descriptions between the unbiased and the strongly-biased translocation [25]. The scaling pictures of polymer translocation now become more clear and complete. The predicted exponents were α=1+ν and δ=1 for both of the weakly-driven and the strongly-driven translocations, whereas α=2+ν and δ=0 for the unbiased translocation [25].

To verify the above theories, the scaling behavior of polymer translocation has been studied intensively by simulations, including Monte Carlo types [26,27,28,29,30,31,32,33,34,35] and molecular dynamics types of study [36,37,38,39,40,41,42,43,44,45,46,47,48]. The situation is similar to the one in the theoretical analyses: the reported exponents are scattered. The value of α falls mainly in a range between one and 1.7 for biased translocation and between 2.2 and 2.6 for the unbiased one in the simulations of three-dimensional space. The exponent δ was mostly found to be close to one. Readers can refer to the review papers [8,9] for a comparison of the reported values. The causes of the non-consistencies were mainly attributed to the modelings, focusing on the finite chain length effect [49,50,51], the pore size [50,52,53,54], the friction or the interaction with the pore [34,55,56,57,58,59], the monomer crowding [23] and the viscosity or the quality of the solvent [50,60,61,62,63,64]. The impact of the hydrodynamic interaction on the behavior of translocation was investigated by simulations as well [65,66,67]. How the kinetics of polymer translocation is influenced by an out-of-equilibrium initial configuration has been discussed recently [68]. The role of chain stiffness on driven translocation was analyzed; the scaling regimes have been classified into the rigid-chain (α=2), the Gaussian-chain (α=1.5) and the excluded-volume chain (α=1+ν) regimes and verified by simulations [69,70]. To understand tension propagation on a chain, two-dimensional intensity maps of the tensile force have been studied in the translocation simulations [47,71,72,73]; the calculations involved, at the same time, the study of the variations of the local monomer velocity, the bond length, the monomer-to-pore distance, etc.

In the simulations, the majority of the works investigated translocation using neutral chains as the studying models. Only a few papers used charged chains with explicit ions to explore the dynamics of translocation [48,72,74,75,76,77]. As we know, the biomacromolecules concerned in the applications of translocation, such as DNA, RNA and proteins, are mostly ionizable molecules in aqueous solutions and belong to a general category of linear polymer, called “polyelectrolyte”. The presence of the electrostatic interaction and the mobile ions in the systems enormously increases the difficulty in treating the problems and results in many astonishing correlations and complicated cooperative behaviors [78,79,80]. Therefore, more theoretical efforts should be invested in the understanding of polyelectrolyte translocation to explore this relatively less-developed field. With modeling the ions, the blockade of ionic current during the moment of chain translocation has been investigated [76,81]. Decreasing the size of counterions was found to slow down the DNA translocation [82]. Recently, we performed a detailed translocation study, using polyelectrolytes in the simulations [48,72]. The scaling behavior of translocation time, the conformational change of the chain, the condensation of ions, the distribution of monomers on the cis and trans sides, the tension propagation, the waiting time function and the drift-diffusion properties have been analyzed.

This paper extends our previous work to simulate driven translocation of polyelectrolyte in the presence of divalent salts. The motivation comes from the technical problem encountered in DNA translocation concerning the high translocation speed [83]. Adding multivalent counterions in the solutions, such as divalent ones, can reduce the effective charge of DNA molecules and thus slow down the process, which renders the detection more feasible and accurate [84,85,86]. Inspired by the work of Sakaue [25], we investigate the situations with the driving force varying from a negligibly weak force to a very strong force, and the scaling behavior is studied by changing the chain length. For comparison, the translocation in the monovalent salt solution is revisited, to study the complete scaling behaviors by covering the entire force regimes. We first rederive the scaling theory in Section 2. Attention is paid to clarifying some of the scaling exponents, which were mixed in Sakaue’s derivation [25]. The predicted scalings are then verified by performing Langevin dynamics simulations. Section 3 describes the model and the settings of the simulations. The results are presented in Section 4. The scalings of the mean translocation time are studied by varying separately the driving field *E* and the chain length *N*. The exponents α and δ are calculated and plotted as a function of *E* and *N* in the monovalent and divalent salt solutions. We further calculate the exponent $\nu_{s0}$ for the size of tethered chains and $\nu_{b0}$ for the one of chain blobs; the diffusion exponent $\gamma_{p}$ is studied as well. Comparisons with the theoretical predictions are made. The possible reasons for the discrepancies are given and discussed. The simulation results are further compared with the experimental data reported in the literature. We give our conclusions in Section 5.

## 2. Scaling Theory

Consider the problem of a polymer translocating across a thin membrane through a nanopore. The polymer comprises *N* monomers. For starting, the body of the chain is placed on the left-hand side of the membrane (called the “cis region”) with the head monomer traversing the pore and locating just at the exit of the pore. We assume that there is a potential barrier in the pore, which acts only on the head monomer to prevent it from reentering to the pore. Therefore, the retraction of the entire chain into the cis region due to the entropic pulling of the chain body will not occur.

A driving force *f* is exerted inside the pore and drives the chain, one monomer by one monomer, through the pore to the right-hand side of the membrane, called the “trans region”. The dynamics of the translocation depends on *f*. Following the work of Sakaue [25], we rederive the scaling behaviors of translocation in different force regimes: the unbiased regime, the weakly-driven regime and the strongly-driven regime. The strongly-driven regime is further divided into the trumpet regime and the isoflux regime.

### 2.1. Unbiased Regime

**Figure 1:**
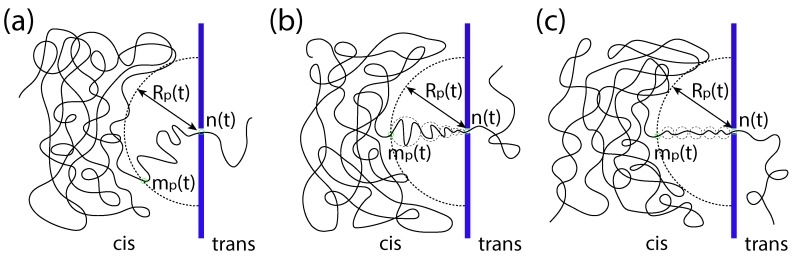
(**a**) A sketch illustrating the translocation coordinate *n*, the position of the tension front $R_{p}$ and the index of the monomer at the tension front, $m_{p}$, at time *t*. (**b**) An illustration for the strongly-driven trumpet (SD(T)) force regime, showing the formation of the trumpet-like shape of chain segments between the tension front and the pore. (**c**) A drawing showing the formation of the isofluxed chain segments in the strongly-driven isoflux (SD(I)) force regime.

A translocation process is said to be in the unbiased (UB) regime if the driving force *f* is negligibly small. In this regime, the translocation happens just because of a random walk. When the process begins, an imbalanced tension appears at the pore entrance and propagates along the cis-side chain, toward the tail end. Let $m_{p}\left(t\right)$ be the index of the monomer at which the front of the tension locates at time *t*. Beyond $m_{p}\left(t\right)$, the monomers have not yet been influenced by the tensile force. Thus, the position of the front can be estimated by the static monomer position $R_{p}\left(t\right)∼m_{p}{\left(t\right)}^{\nu_{s}}$, where $\nu_{s}$ is the exponent, which describes the scaling of the equilibrium distance of the $m_{p}$-th monomer to the first monomer on a chain tethered on the surface. To describe the progress of translocation, a variable n(t), called the translocation coordinate, is defined. It counts the number of the monomers having been translocated into the trans region at time *t*. A sketch to illustrate the variables $R_{p}$, $m_{p}$ and *n* of translocation is given in Figure 1a.

The translocation process is divided into two stages: (1) the tension propagation stage, which happens before the arrival of the tension front at the chain tail, and (2) the post-propagation stage, which describes the moment after the arrival of the front at the chain end. The translocation time τ is the sum of the time $\tau_{p}$ and $\tau_{\mathrm{pp}}$, respectively, in the two stages. In the tension propagation stage ($t\leq \tau_{p}$), a dynamical exponent $z_{p}$ is introduced to describe the scaling of the propagation: $t∼R_{p}^{z_{p}}$. It yields $\tau_{p}∼N^{\nu_{s}z_{p}}$ because at $t=\tau_{p}$, the tension front arrives at the chain end, and hence, $m_{p}=N$. In this stage, the process shows a significant memory effect [15,17,19,25,87]. The dynamics is subdiffusive, and $\langle \Delta n^{2}\left(t\right)\rangle$ scales as $t^{\gamma_{p}}$ where $\gamma_{p}$ is the subdiffusion exponent. It was argued that $\gamma_{p}=\left(1+\nu_{s}\right)/\left(z_{p}\nu_{s}\right)$ (see Appendix A). We remark that there exists another dynamical exponent *z*, describing the time scaling of diffusion of a chain blob over its size ξ in a solution through the relation $\tau_{\xi }∼\xi^{z}$ [88]. The exponent can be related to the size exponent $\nu_{b}$ of a blob via the equation $z-2=1/\nu_{b}$ in Rouse dynamics [18,25]. Please do not confuse it with $z_{p}$, which describes the time scaling of a tension propagating on a chain [89].

In the post-propagation stage, the dynamics becomes a normal diffusion process: $\langle \Delta n^{2}\left(t\right)\rangle ∼D_{n}t$. The continuity of the $\langle \Delta n^{2}\left(t\right)\rangle$ curve at $t=\tau_{p}$ gives the opportunity to estimate the diffusion coefficient $D_{n}$, which scales as $\tau_{p}^{\gamma_{p}-1}$. We hence have $D_{n}∼N^{\nu_{s}z_{p}\left(\gamma_{p}-1\right)}$. Let $Q_{N}\equiv \sqrt{\langle \Delta n^{2}\left(\tau_{p}\right)\rangle }/N$ be the fraction of chain translocated in the tension propagation stage. For the case of a long chain, $Q_{N}$ is anticipated to be much smaller than one. We thus have $\nu_{s}z_{p}\gamma_{p}<2$, and the translocation time is dominated by the post-propagation time. Therefore, $\tau ≃\tau_{\mathrm{pp}}∼{\left[N\left(1-Q_{N}\right)\right]}^{2}/D_{n}∼N^{2+\nu_{s}z_{p}\left(1-\gamma_{p}\right)}$.

### 2.2. Weakly-Driven Regime

The driving force starts to manifest its effect since in the weakly-driven (WD) regime, and the translocation shows drift-diffusion behavior. In the tension propagation stage, the drift of the chain is anomalous and can be written as $\langle n(t)\rangle ∼ft^{\gamma_{p}}$, while in the post-propagation stage, it becomes a normal drift, described by $\langle n(t)\rangle ∼\frac{f}{\Gamma_{n}}t$. Here, $\Gamma_{n}=k_{B}T/D_{n}$ is the friction coefficient, *T* is the temperature and $k_{B}$ is the Boltzmann constant. The post-propagation time $\tau_{\mathrm{pp}}$ can be calculated by $N\left(1-Q_{N}\right)\Gamma_{n}/f$. If the chain length is long, the fraction $Q_{N}=\langle n(\tau_{p})\rangle /N$ of the translocated chain in the first stage is negligible. The translocation time is again dominated by the post-propagation time. We have $\tau ≃\tau_{\mathrm{pp}}∼N^{1+\nu_{s}z_{p}\left(1-\gamma_{p}\right)}f^{-1}$.

The boundary that distinguishes the UB and the WD regimes can be estimated from the inequality: $\tau_{\mathrm{UB}}≳\tau_{\mathrm{WD}}$, and yields $f≳N^{-1}$. The two force regimes are hence demarcated at $f_{1}^{*}∼k_{B}T/\left(Nb\right)$ where *b* is the bond length of the chain.

### 2.3. Strongly-Driven Regime

In the strongly-driven (SD) regime, the tension propagation constitutes the major part of the process. The translocation time is hence approximately $\tau_{p}$. In this regime, a series of tension blobs is formed along the pore axis of the system (called the *x*-axis), between the tension front and the pore entrance. One can refer to Figure 1b for an illustration. The blob size, $\xi_{x}∼k_{B}T/f_{x}$, depends on the tension force $f_{x}$ on the chain at the position *x*. We assume that a blob comprises $g_{x}$ monomers and the size of the blob is $\xi_{x}∼g_{x}^{\nu_{b}}$, where $\nu_{b}$ is the scaling exponent of a chain blob in a free solution. The line density of the monomer along the *x*-axis is thus $\sigma_{x}=g_{x}/\xi_{x}∼\xi_{x}^{(1/\nu_{b})-1}$. The moving velocity of the blob can be computed from the local balance equation $v_{x}=f_{x}/\Gamma_{x}$ and thus scales as $\xi_{x}^{-1-(1/\nu_{b})}$; here, the friction coefficient $\Gamma_{x}$ of the blob is proportional to $g_{x}$ in Rouse dynamics. Therefore, we have the scaling $\sigma_{x}∼v_{x}^{-q}$ with $q=\left(1-\nu_{b}\right)/\left(1+\nu_{b}\right)$, which relates the local monomer density with the blob moving velocity.

The dynamics of the process can be then studied via the rate equation of change for the number of the monomers within the tension front:

(1) $$\frac{d}{dt}\left(m_{p}-n\right)=j_{p}-j_{0}+\sigma_{p}\frac{dR_{p}}{dt}$$

where $j_{p}$ and $j_{0}$ are the fluxes of the monomers across the tension front and the pore, respectively, and $\sigma_{p}$ is the linear density of monomer at the front. The equation is reduced to:

(2) $$\frac{dm_{p}}{dt}=\sigma_{p}\left[v_{p}+\frac{dR_{p}}{dt}\right],$$

because $j_{p}=\sigma_{p}v_{p}$ and $j_{0}=dn/dt$. Extending the idea of Brochard-Wyart [90,91,92], a velocity-extension- force relation is presumed under the form $v_{p}R_{p}∼f^{p_{z}}$. It was argued that the exponent $p_{z}$ is z−2 [93]. Using the velocity-extension-force relation, together with the scalings $m_{p}∼R_{p}^{1/\nu_{s}}$ and $\sigma_{p}∼v_{p}^{-q}$, to solve Equation (2) for $R_{p}\left(t\right)$, we obtained $R_{p}^{(1/\nu_{s})-q+1}-R_{p}^{2}f^{-p_{z}q}∼tf^{p_{z}\left(1-q\right)}$. The second term on the left is negligible for large *f*. By setting $t=\tau_{p}$ and $R_{p}∼N^{\nu_{s}}$, we have $\tau_{p}∼N^{1+\nu_{s}\left(1-q\right)}f^{-p_{z}\left(1-q\right)}$. It is approximately the translocation time.

This force regime is called “the trumpet force regime” because the ensemble of the series of the tension blobs formed on the cis side looks like a trumpet. We denote it by SD(T). The lower boundary of the SD(T) regime can be found by equating the translocation time with the one in the WD regime. It yields $f_{2}^{*}∼N^{-\nu_{s}\rho }$ where $\rho =\left(z_{p}\left(1-\gamma_{p}\right)-1+q\right)/\left(p_{z}\left(1-q\right)-1\right)$.

If the driving force grows even higher, the system can enter into another regime called “the isoflux force regime”, denoted by SD(I). In this regime, the flux of monomers within the tension front is constant. The blob size $\xi_{x}$ and velocity $v_{x}$ are thus independent of *x*. Figure 1c illustrates this situation. The translocation velocity is described by the equation:

(3) $$\frac{dn}{dt}∼v_{0}∼\frac{f}{m_{p}-n}.$$

Using $R_{p}∼m_{p}-n$ to solve the equation for $R_{p}$, we got $R_{p}^{(1/\nu_{s})+1}-R_{p}^{2}∼ft$. The first term on the left-hand side is the dominated term. It gives $\tau_{p}∼N^{1+\nu_{s}}f^{-1}$, which is approximately the translocation time. The boundary between the SD(T) and SD(I) regimes is found to locate at $f_{3}^{*}∼N^{-\nu_{s}\eta }$ where $\eta =q/(p_{z}\left(1-q\right)-1)$.

### 2.4. Summary of the Scaling Behaviors

The obtained results are summarized below:

(4) $$\begin{array}{c} \tau =\left\{\begin{array}{c} \tau_{\mathrm{UB}}∼N^{2+\nu_{s}z_{p}\left(1-\gamma_{p}\right)}\\ \tau_{\mathrm{WD}}∼N^{1+\nu_{s}z_{p}\left(1-\gamma_{p}\right)}f^{-1}\\ \tau_{\mathrm{SD}(T)}∼N^{1+\nu_{s}\left(1-q\right)}f^{-p_{z}\left(1-q\right)}\\ \tau_{\mathrm{SD}(I)}∼N^{1+\nu_{s}}f^{-1} \end{array}\right. \end{array}$$

**Figure 2:**
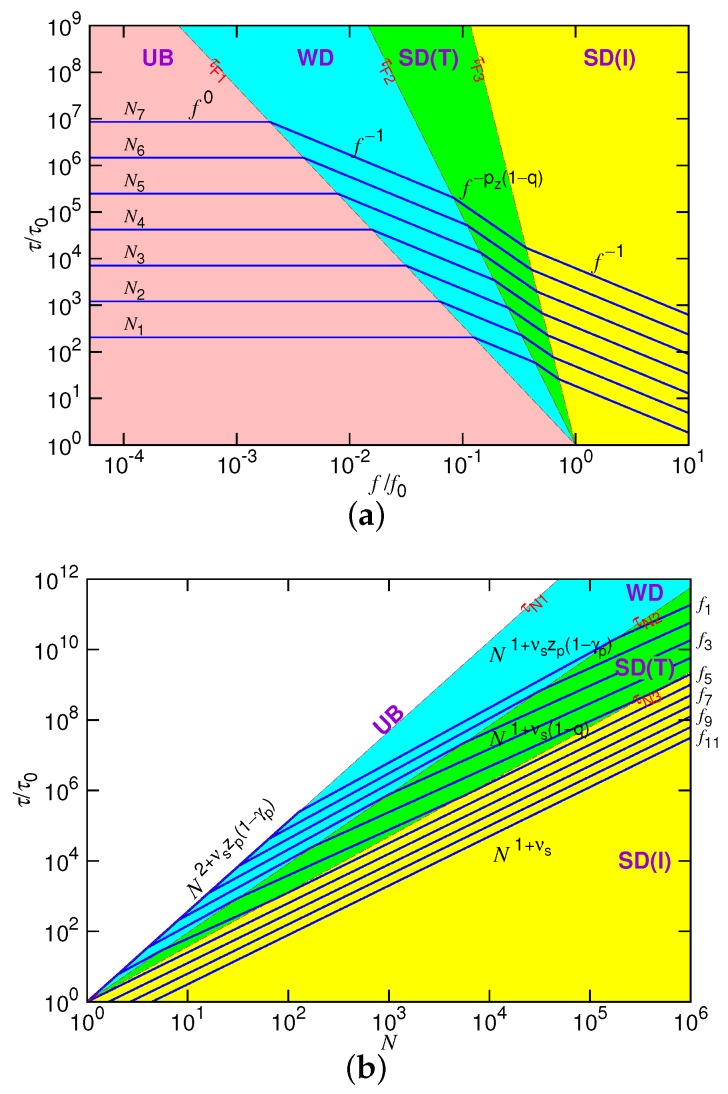
(**a**) Translocation time τ vs. *f* at different chain lengths *N* and (**b**) τ vs. *N* at different driving forces *f*. In the plots, we set $\nu_{s}=0.4$, $z_{p}\left(1-\gamma_{p}\right)=1.4$, $p_{z}\left(1-q\right)=1.67$ and q=0.25. In (a), $N_{i+1}=2N_{i}$ with $N_{1}=8$; in (b), $f_{i+1}=2f_{i}$ with $f_{1}=1/128.$ UB, unbiased; WD, weakly-driven.

The translocation time τ shows four scaling behaviors in the different force regimes separated by the three boundaries $f_{1}^{*}$, $f_{2}^{*}$ and $f_{3}^{*}$ at a given chain length *N*. The plot of τ against *f* is presented in Figure 2a in the log-log scales. The four force regimes are demarcated by the three lines: $\tau_{F1}∼f^{-X_{1}}$, $\tau_{F2}∼f^{-X_{2}}$ and $\tau_{F3}∼f^{-X_{3}}$, where $X_{1}=2+\nu_{s}z_{p}\left(1-\gamma_{p}\right)$, $X_{2}=\left(\left(\nu_{s}^{-1}+1-q\right)/\rho \right)+p_{z}\left(1-q\right)$ and $X_{3}=\left(\left(\nu_{s}^{-1}+1-q\right)/\eta \right)+p_{z}\left(1-q\right)$. To have the well-defined four regimes in the long-chain limit, these exponents should satisfy the condition $X_{1}<X_{2}<X_{3}$, which implies ρ>η and $\nu_{s}\rho <1$. To see how “τ vs. *f*” changes with *N*, we have plotted several curves in the figure by doubling the chain length of each curve in the way $N_{i+1}=2N_{i}$. The curves move upward with increasing *N*, obviously showing that a longer translocation time is required by a longer chain. We, moreover, observed a diminishing of the UB force regime with increasing of the chain length.

Figure 2b presents the predicted scaling behavior of τ versus *N* at fixed driving forces *f*. For *N* smaller than $N_{1}^{*}∼f^{-1}$, the force is negligible for the system and the translocation is unbiased. The chain situates in the weakly-driven condition when *N* lies between $N_{1}^{*}$ and $N_{2}^{*}∼f^{-1/(\nu_{s}\rho )}$. The trumpet (SD(T)) and the isoflux (SD(I)) force regimes can be shown to be separated by $N=N_{3}^{*}∼f^{-1/(\nu_{s}\eta )}$.

In the figure, the force regimes are demarcated by the three lines: $\tau_{N1}∼N^{Y_{1}}$, $\tau_{N2}∼N^{Y_{2}}$ and $\tau_{N3}∼N^{Y_{3}}$ with $Y_{1}=2+\nu_{s}z_{p}\left(1-\gamma_{p}\right)$, $Y_{2}=1+\nu_{s}z_{p}\left(1-\gamma_{p}\right)+\nu_{s}\rho$, and $Y_{3}=1+\nu_{s}+\nu_{s}\eta$. The slopes of the lines satisfy the order $Y_{1}>Y_{2}>Y_{3}$, if $p_{z}\left(1-q\right)>1$ and $z_{p}\left(1-\gamma_{p}\right)>1$. Please notice that the UB regime shrinks completely and, thus, is just a line in the figure. This is because $\tau_{\mathrm{UB}}$ and $\tau_{N1}$ have the same exponent when scaling with *N*. To show the influence of the driving force, we have drawn the τ curve versus *N* by doubling *f* in the way $f_{i+1}=2f_{i}$. In each force regime, the time curves show parallel lines. Sine ρ>0, the exponent of the curve in the WD regime is larger than the one in the SD(T) regime. Therefore, the gap of the parallel lines increases when the curves enter from the WD to the SD(T) regime. When entering later into the SD(I) regime by increasing *N*, the exponent increases. The gap of the lines is thus narrowed down.

To verify the scaling theory presented here, molecular dynamics simulations were performed in this study. The results and discussions are given in the following sections.

## 3. Simulation Model and Setup

We performed molecular dynamics simulations to study single polyelectrolytes threading through a nanopore. The polyelectrolyte was modeled by a charged bead-spring chain, which comprises $N_{m}$ monomers. Each monomer carries a negative unit charge −e and dissociates a monovalent cation into the solution. A fixed amount of (*Z*:1)-salt molecules was added into the system. A salt molecule dissociates into one +*Z*-cation and *Z* monovalent anions in the solution. A wall was placed in the middle of the simulation box. It divided the system into two subspaces in the *x*-direction, called the cis and the trans regions, respectively. The two regions are connected by a pore drilled through the wall. Inside the pore, a uniform electric field was applied. The wall was built up by beads, and the beads were set immobile to save the resources of computation. The thickness of the wall was 4.5σ, and the radius of the pore was 2.25σ, where σ is the length unit of simulation. The simulations were performed in a rectangular box. The three sides of the box were $L_{x}=200.0\;\sigma$, $L_{y}=48.0\;\sigma$, $L_{z}=49.4\;\sigma$. The periodic boundary condition was employed.

The excluded volume interaction of bead was modeled by the Weeks–Chandler–Andersen (WCA) potential [94],

(5) $$\begin{array}{c} U_{\mathrm{ex}}\left(r_{ij}\right)=\left\{\begin{array}{cc} \varepsilon_{ij}{\left[2{\left(\frac{\sigma_{ij}}{r_{ij}}\right)}^{6}-1\right]}^{2} & \mathrm{for}\;r_{ij}\leq \sqrt[{6}]{2}\sigma_{ij}\\ 0 & \mathrm{for}\;r_{ij}>\sqrt[{6}]{2}\sigma_{ij} \end{array}\right. \end{array}$$

where $\varepsilon_{ij}$ and $\sigma_{ij}$ are, respectively, the interaction strength and the distance between two particles *i* and *j*. We set $\sigma_{\mathrm{pp}}=1.0\;\sigma$ and $\varepsilon_{\mathrm{pp}}=1.2\;k_{B}T$ for the interactions between the mobile particles (p), including the monomers and the ions. The interactions between the mobile particles and the immobile wall beads (w) were set to be $\sigma_{\mathrm{pw}}=1.5\;\sigma$ and $\varepsilon_{\mathrm{pw}}=2.5\;k_{B}T$. Here, the thermal energy $k_{B}T$ is used as the energy unit. The electrostatic interaction between a pair of charged beads was given by:

(6) $$U_{\mathrm{el}}\left(r_{ij}\right)=k_{B}T\lambda_{B}\frac{Z_{i}Z_{j}}{r_{ij}}$$

where $Z_{i}$ and $Z_{j}$ are the valences of the beads and $\lambda_{B}$ is the Bjerrum length, which describes the coupling strength of the electrostatics in the solution. We set $\lambda_{B}=3.0\;\sigma$ and calculated the electrostatic interactions by the particle-particle particle-mesh Ewald method [95,96,97]. The adjacent monomers on the chain were connected by a harmonic bond

(7) $$U_{\mathrm{bd}}\left(b_{ij}\right)=\frac{1}{2}k{\left(b_{ij}-b_{0}\right)}^{2}$$

with the spring constant $k=600.0\;k_{B}T/\sigma^{2}$ and the equilibrium bond length $b_{0}=1.0\;\sigma$. The mass of a mobile bead is *m*.

Initially, a chain was equilibrated by constraining the head monomer at the exit of the pore on the trans side with the chain body, traversing the pore, locating mainly in the cis region. A translocation process was started by removing the constraint and, at the same time, switching on the electric field $\vec{E}=-E\hat{x}$ in the pore. The electric field drove the negatively-charged monomers toward the $+\hat{x}$ direction, and the chain was transported, one monomer by one monomer, via the pore, from the cis side to the trans side of the system. To prevent the retraction of the entire chain back into the cis region, a potential barrier was set at the pore exit, acting only on the head monomer to prohibit it from returning to the pore. The probability for a failed translocation process to occur [98] and the rate of capturing a polymer by a pore [99] are not concerned in this study. Other effects such as the charged wall, the electroosmotic flow, the funneling electric field exhibited outside the pore and the varying of the electric field distribution due to the passing of chains and ions [7] are not considered.

We studied two salt solutions: one was of the monovalent salt (Z=1), and the other was of the divalent salt (Z=2). The amount of the adding salt was 256 molecules in both cases. The field strength was varied from E=0.001 to $64.0\;k_{B}T/\left(e\sigma \right)$, which spans over five orders of magnitude of the field strength, covering from very weak electric fields to very strong fields. The chain length was varied from $N_{m}=8$ to 384. The temperature was controlled by a Langevin thermostat [100,101,102] with the damping time set to $1.0\;t_{u}$ where $t_{u}=\sigma \sqrt{m/k_{B}T}$ is the simulation time unit. For each set of the simulation parameters (*Z*, $N_{m}$, *E*), at least 500 independent runs were performed. The data were collected and analyzed statistically. More information about the modelings and the settings can be found in our previous paper [48].

In the following text, σ, *m*, $k_{B}T$, *e* will be used as the length, the mass, the energy, the charge units, respectively, to describe or report the data. To shorten the notation, we will give only the value of a physical quantity and omit the unit. For example, the field strength “E=0.2” in the text stands for $E=0.2\;k_{B}T/\left(e\sigma \right)$, and the translocation time “τ=100” means $\tau =100\;t_{u}=100\;\sigma \sqrt{m/k_{B}T}$.

**Figure 3:**
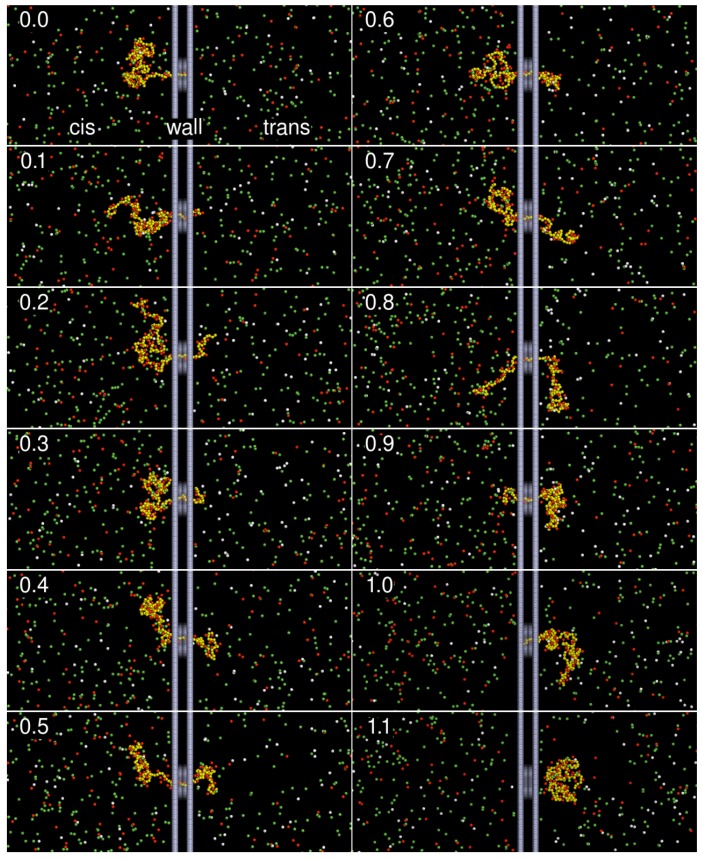
Snapshots of a threading process. The polyelectrolyte is represented by a yellow bead-spring chain. The divalent counterions, monovalent counterions and coions are represented in the red, white and green beads, respectively. A hallow wall separates the space into the cis and trans regions, connected by a pore channel at the center. A driving electric field $\vec{E}=-E\hat{x}$ is applied inside the pore. In the snapshots, the chain length is $N_{m}=128$, and *E* is 0.5. The number on the left-top corner of each snapshot indicates the progress of the translocation, defined as the ratio of the elapsed time over the translocation time. To visualize the chain inside the pore, the wall beads (in gray color) have been plotted with certain degree of transparency.

To have an illustration of how the system is in a translocation process, the snapshots of a simulation run in the divalent salt solution are given in Figure 3. The chain comprises $N_{m}=128$ monomers, driven by an electric field E=0.5 inside the pore. The number printed on the left-top corner of each snapshot is the ratio t/τ, which indicates the progress of translocation, where *t* is the elapsed time and τ is the translocation time. The red and white beads represent the divalent counterions ((+2)-ions) and monovalent counterions ((+1)-ions), respectively, while the coions ((−1)-ions) and the monomers are represented by the green and yellow beads. We can see that considerable divalent counterions were condensed on the chain during the translocation process. Some of these ions can be even dragged through the pore with the translocated chain.

## 4. Results and Discussion

A nanopore has always a finite length in reality. Therefore, there must be a small amount of time spent for the last few monomers to traverse the pore at the last moment of chain translocation. This amount of time becomes important if the chain is not long in comparison with the pore length. There is another important fact to be considered: the nature of translocation is drift-diffusive. The monomers of the chain can go back and forth momentarily inside the pore and visit some place in the pore several times, particularly when the driving force is weak. To perform the study properly by diminishing the impact of the two facts, we defined the translocation time τ to be the time needed for a chain to definitely leave the cis side in a translocation process. This definition, compared with the common definition using the first passage time to determine the translocation time [7,8,9], takes into account the diffusive nature of the problem. Moreover, in our simulations, the translocation was started with the first five monomers initially spanned across the pore. The exact number of monomers transported from the cis side was thus not $N_{m}$, but $N=N_{m}-5$.

### 4.1. Mean Translocation Time in the (1:1)-Salt Solution

**Figure 4:**
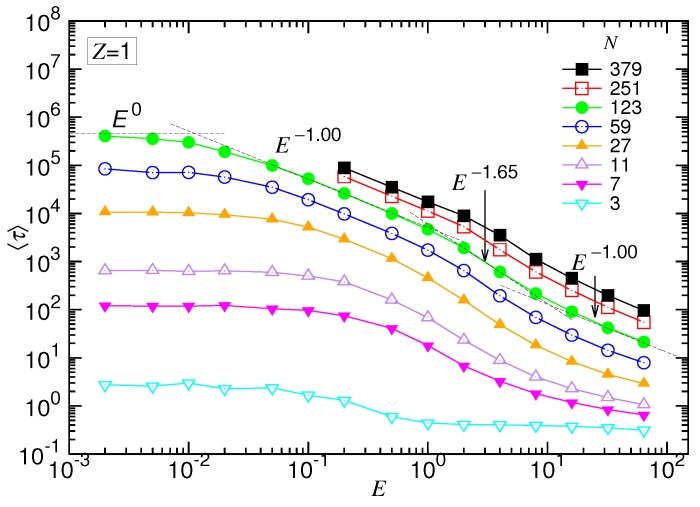
Mean translocation time 〈τ〉 as a function of the driving field *E* in the monovalent salt solution. The value of *N* can be read in the legend. The dashed lines show the four predicted scaling behaviors for N=123.

The translocation time τ was studied systematically by varying the strength of the driving field *E* and the number of monomers *N*. The mean value was presumed to exhibit the scaling: $\langle \tau \rangle ∼N^{\alpha }E^{-\delta }$. We first studied the behavior of 〈τ〉 as a function of *E* at fixed *N*. Figure 4 presents the results in the (1:1)-salt solution.

We can see that the time was about constant when the driving field was very weak. As *E* increased over some value, 〈τ〉 decreased and showed, in turn, three scaling behaviors. For the case of N=123, the scaling exponent δ changed from 1.0 to 1.65 and then regained the value 1.0. The results support the prediction of the scaling theory. The translocation was apparently separated into four regimes, corresponding to the UB, WD, SD(T) and SD(I) force regimes, respectively.

**Figure 5:**
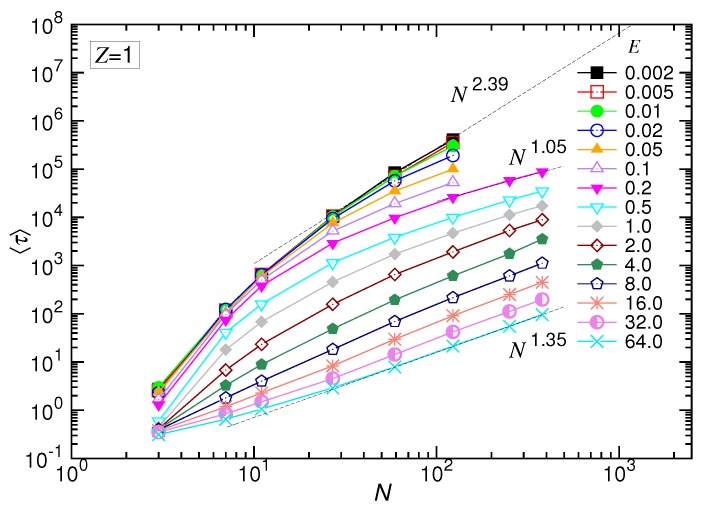
Mean translocation time 〈τ〉 as a function of the number *N* of the monomers transported in the monovalent salt solution. The strength of the driving field *E* can be read from the legend. The dashed lines indicate the three characteristic scaling behaviors.

Figure 5 shows the behavior of 〈τ〉 by varying *N* at fixed *E* fields. The variations of the curves look quite similar to the predicted behavior given in Figure 2b, except for the appearance of a faster shrinkage of the set of the curves for *N* smaller than 20. The occurrence of the faster shrinkage is not a surprise because the scaling theory is derived in the long-chain limit. For short chains, the chain conformation was generally rod-like, which is less coiled than for long chains. Thus, transporting a short chain through a pore suffered weaker resistance than a long chain. The translocation time was consequently shortened in weak fields. For the strong driven cases, the second effect dominated: a non-negligible fraction of time was spent, at starting, on confronting the inertia of the chain. This amount of time was not considered in the derivation. Therefore, 〈τ〉 was longer than the predicted value. Combining the two effects, the set of the curves converged faster than the predicted behavior as *N* was small.

The scaling theory stated that the translocation time was upper bounded by a UB line given by $N^{\alpha }∼N^{2+\nu_{s}z_{p}\left(1-\gamma_{p}\right)}$. By increasing the driving force, the exponent α decreased by one as the system entered the WD regime. It then changed to $1+\nu_{s}\left(1-q\right)$ in the SD(T) regime and, finally, increased to $1+\nu_{s}$ in the SD(I) regime. Our simulations did show the existence of such an asymptotic UB line, which scaled as $N^{2.39}$ extracted by a least-squares fit from the last three data at N=27, 59 and 123. Increasing *E* reduced τ at a given *N*, and the scaling exhibited the characteristic variations: α decreased first and then increased. We studied the changes of the exponent. The exponent extracted from the data N=123, 251 and 379 gave a value of 1.05 for the SD(T) regime and 1.35 for the SD(I) regime, as shown in the figure.

A similar variational behavior of 〈τ〉 against the force *f* had been reported in the previous study of translocation using neutral polymers pulled from the head end [103,104]; the systems were found to be driven from a unbiased condition, in which 〈τ〉 was constant, to a biased one, in which 〈τ〉 decreased with increasing the pulling force. The asymptotic UB scaling line in the log-log plot of 〈τ〉 vs. *N* had been seen in the study using neutral chains as well [34]. The curves showed less variational structure, compared with the results obtained here using polyelectrolytes.

**Figure 6:**
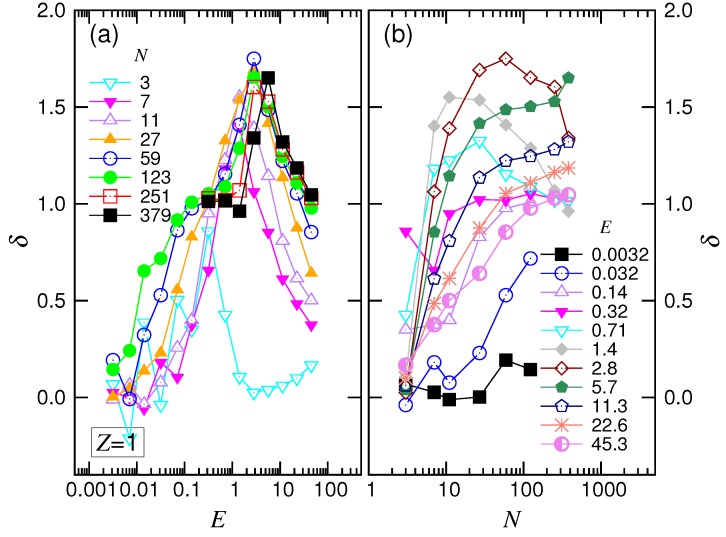
Exponent δ (**a**) as a function of *E* at different *N* and (**b**) as a function of *N* for different *E*, in the monovalent salt solution. The values of *E* in the plots are the geometric means of the pairs of the adjacent driving field strengths given in the legend of Figure 5.

**Figure 7:**
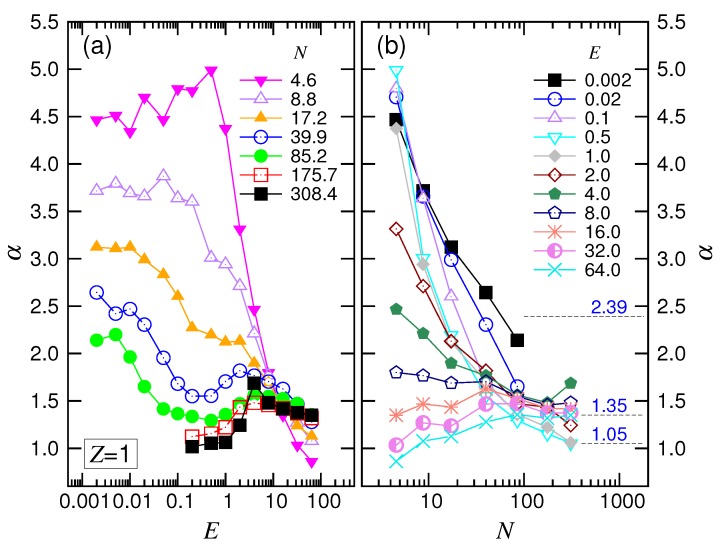
Exponent α (**a**) as a function of *E* at different *N* and (**b**) as a function of *N* for different *E*, in the monovalent salt solution. The values of *N* in the plots are the geometric means of the adjacent *N* pairs given in the legend of Figure 4.

To understand how δ and α vary with *E* and *N*, we calculated the scaling exponents from pairs of the adjacent data on the curves in the above two figures. The results are presented in Figure 6 and Figure 7.

δ is about zero at very small *E* in Figure 6a, showing that the system was in the UB regime. For long chains, the value of δ increased with increasing *E* and arrived at a plateau value of around one in the middle range of *E*. The system was the WD force regime. Passing the plateau, the exponent climbed again and exhibited a peak of height about 1.65. This was characteristic of the SD(T) behavior. Afterward, δ decreased and tended to reach the value of one at the extremely large driving fields. The system had now arrived at the SD(I) regime. The value of δ was seen to be smaller than one for short chains. This is probably because the chains were not long enough to allow the translocation to enter the assumed “isoflux” condition, resulting in the deviation from the predicted SD(I) behavior.

For the variation against *N* in Figure 6b, δ stayed around zero at the beginning in the weak driving fields E≤0.032 and switched to increasing with *N*. Thus, the longer the chain, the easier for the chain to leave the UB regime. In the intermediate fields, δ rose and descended, exhibiting a maximum. The exponent returned to an increasing function when *E* was stronger than 5.7.

The exponent α depended on both *E* and *N* as well. Figure 7a shows that α stayed on a plateau value at small *E*. The plateau shifted downward as *N* increased and tended toward a value around 2.4. As *E* increased for long chains, α first decreased its value by one; it then increased and finally decreased. These changes show the four featured behaviors in the force regimes.

For the variation of α with *N* (refer to Figure 7b), we found that α decreased and approached 2.39 at small *E*. At intermediate *E*, the exponent decreased with increasing *N* and converged to a value at about 1.05. For a large driving field E≥4.0, the convergence shifted upward, and the value was around 1.35. According to the scaling theory, our simulation results gave $\nu_{s}=0.35$ and q=0.86. We will discuss these later.

### 4.2. Mean Translocation Time in the (2:1)-Salt Solution

**Figure 8:**
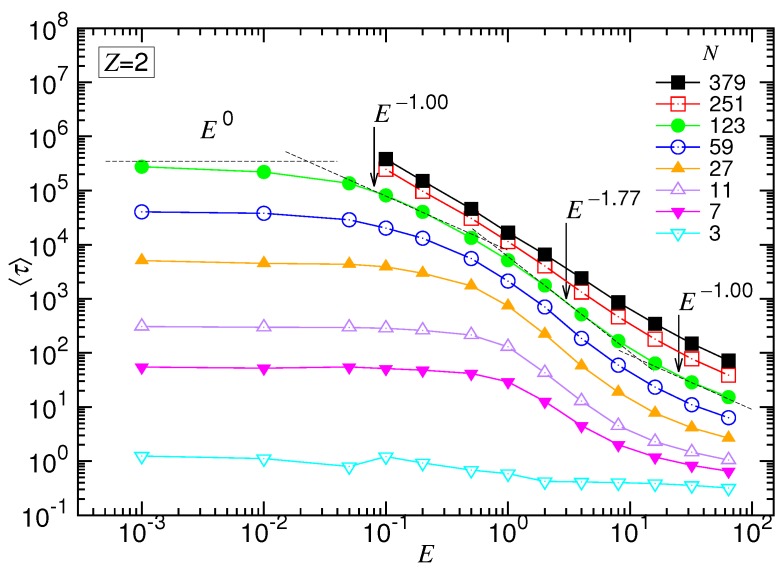
Mean translocation time 〈τ〉 as a function of the driving field *E* in the divalent salt solution. The value of *N* can be read in the legend. The dashed lines indicate the four scaling behaviors for N=123.

**Figure 9:**
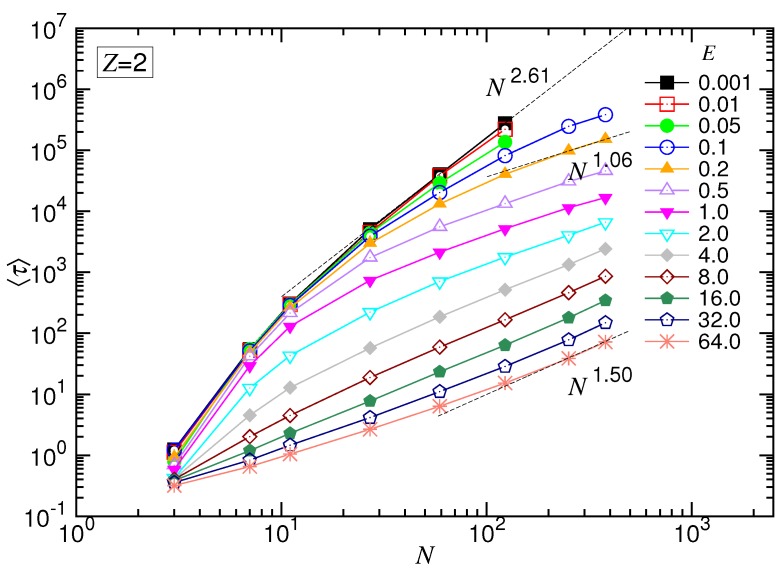
Mean translocation time 〈τ〉 as a function of the number *N* of the monomers transported in the divalent salt solution. The strength of the driving field *E* can be read in the legend. The dashed lines indicate the three characteristic scaling behaviors.

The mean translocation time in the divalent salt solution is presented in Figure 8 and Figure 9, as a function of the driving field *E* at fixed *N* and as a function of *N* at fixed *E*, respectively.

Similar to the monovalent salt, 〈τ〉 vs. *E* exhibited the four characteristic scaling behaviors. For example, at N=123, the exponent δ had the starting value of zero in the UB regime and transited gradually with *E* to 1.0 in the WD regime. It acquired a maximum value 1.77 in the SD(T) regime. Finally, δ diminished to a value around 1.0 at very strong driving fields.

Concerning the dependence of *N*, we observed again the set of the 〈τ〉 curves upper-bounded by a line in the log-log plot, which was $N^{2.61}$. It describes the limiting situation taking place in a negligibly weak driving field, i.e., the UB force regime. By increasing *E* for large *N*, the variation of α exhibited the featured behaviors. It allowed determining the exponent $1+\nu_{s}\left(1-q\right)$ for the SD(T) regime and $1+\nu_{s}$ for the SD(I), which were 1.06 and 1.50, respectively. The results gave $\nu_{s}=0.5$ and q=0.88.

**Figure 10:**
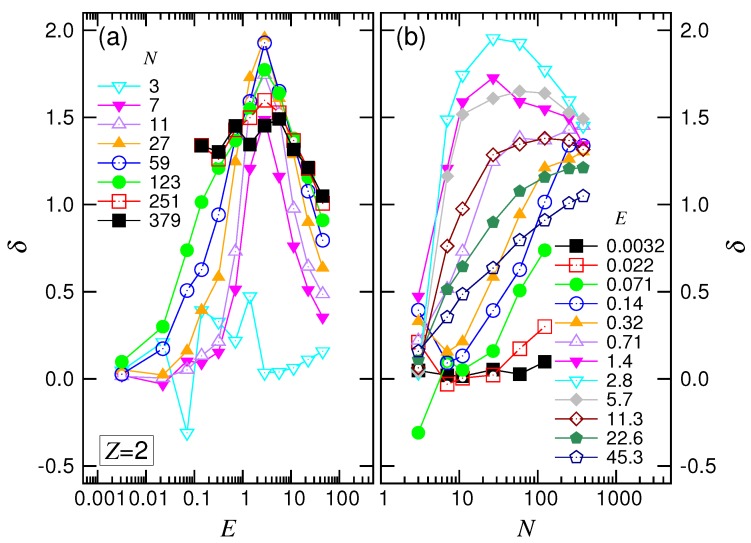
Exponent δ (**a**) as a function of *E* at different *N* and (**b**) as a function of *N* for different *E*, in the divalent salt solution. The values of *E* in the plots are the geometric means of the pairs of the adjacent driving field strengths in the legend of Figure 9.

The exponents δ and α were both functions of *E* and *N*. Figure 10a presented the variation of δ against *E* at fixed *N*. The value of δ started from zero, increased with *E* and attained a peak value at about 1.77. It then decreased and tended to approach one for the long chains.

For the variation of δ against *N* at given *E* shown in Figure 10b, we saw that δ stayed at zero in the weak driving field and turned to increase with *N*. In the intermediate *E*, the δ curve moved upward and exhibited a bump. For large *E*, the curve moved downward and the bump vanished.

**Figure 11:**
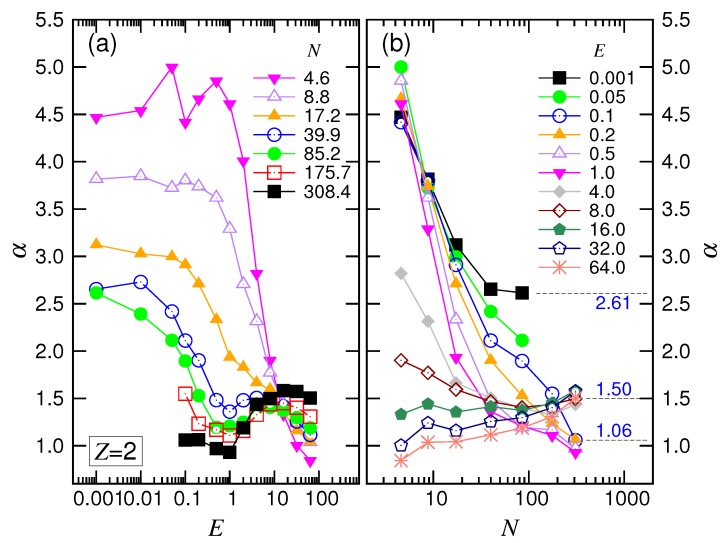
Exponent α (**a**) as a function of *E* at different *N* and (**b**) as a function of *N* for different *E*, in the divalent salt solution. The values of *N* in the plots are the geometric means of the adjacent *N* pairs in the legend of Figure 8.

The variation of α at a given *N* against *E* is plotted in Figure 11a. Similar to the monovalent salt case, α was about a constant in the small *E* region. The constant decreased with increasing *N* and tended to a value of about 2.6. Leaving the constant value by increasing *E*, α was decreased by one, then climbed over a small hill and, finally, arrived at a value of around one. The results show the scaling characteristics of the four force regimes.

In Figure 11b, we saw that α tended toward the value 2.61 with increasing *N* at the small *E* field. In the intermediate range of *E*, α decreased as well, but tended to 1.06. Increasing *E* further decreased α. At large *E*, the exponent turned out to be an increasing function and varied toward 1.50. The scaling behaviors observed in the divalent salt solution were similar to the ones in the monovalent salt solution.

### 4.3. Static Exponents $\nu_{s0}$ and $\nu_{b0}$

**Figure 12:**
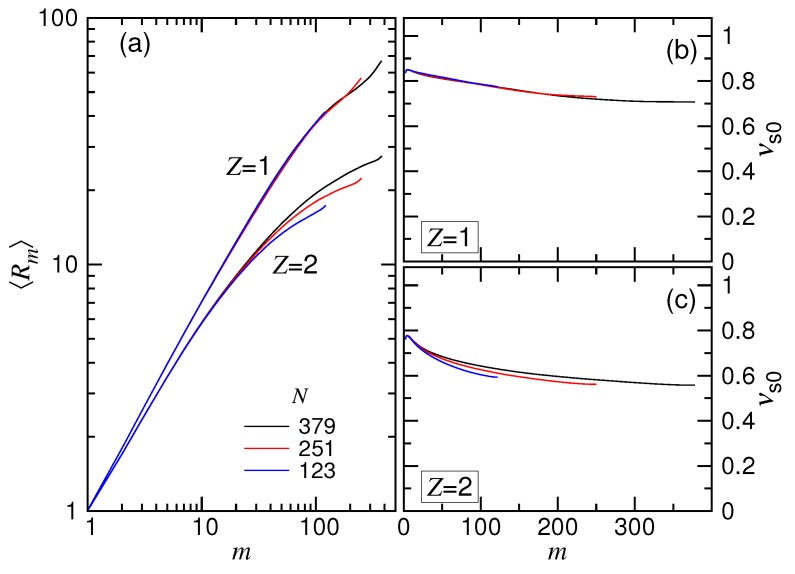
(**a**) Mean distance $\langle R_{m}\rangle$ of a monomer to the tethering point on a tethered chain of length N=123, 251 and 379 in the Z=1 and Z=2 salt solutions. Here, *m* is the monomer index. The calculated exponent $\nu_{s0}$ is plotted against *m* in (**b**) the monovalent and (**c**) the divalent salt solutions.

In the scaling theory, the translocation time has been shown to be related to the exponents $\nu_{s}$ and $\nu_{b}$. The exponent $\nu_{s}$ was introduced to depict the position of a monomer on a chain tethered on a wall through the scaling relation $\langle R_{m}\rangle ∼m^{\nu_{s}}$, where $R_{m}$ is the distance of the *m*-th monomer to the tethering point. To study it, 500 independent simulation runs were performed in the monovalent and the divalent salt solutions for three tethered chain lengths, N=123, 251 and 379. The obtained mean distances $\langle R_{m}\rangle$ are plotted in Figure 12a as a function of *m* where 1≤m≤N.

We saw that the curves were separated into two branches, depending on the valence of the salt, with the Z=1 branch higher than the Z=2 branch. This is because the tethered chain pervaded a larger space in the (1:1)-salt solution than in the (2:1)-salt solution, owing to the weaker screening of Coulomb interaction in the previous. In each branch, because of the effect of finite chain length, the curve for small *N* deviated from the main course as *m* became large.

To study the scaling, we calculated the exponent by the equation $\nu_{s0}=\log\left(\langle R_{m}\rangle /\langle R_{1}\rangle \right)/\log m$. Here, we denote the exponent as $\nu_{s0}$ to emphasize that it is extracted from the simulations with a chain tethered statically on a surface. The exponent $\nu_{s}$ was, on the other hand, obtained from the study of dynamics through the threading-chain simulations shown above. The scaling theory anticipated an equality of the two exponents; however, this was not for sure. The results of $\nu_{s0}$ are presented in Panels (b) and (c) of Figure 12 for the cases Z=1 and Z=2. We can see that $\nu_{s0}$ was not a constant, but depended on *m*. The value was initially 0.85 and 0.78 for the two salt cases and descended with *m* toward 0.71 and 0.57, respectively. When compared with $\nu_{s}=0.35$ and 0.50 from Figure 5 and Figure 9, a significant difference was found. Since $\nu_{s}$ was extracted from the simulations in the SD(I) force regime, it may indicate that the isoflux assumption was not well held in this regime, particularly for the case of Z=1. Other effects such as the crowding of the monomers on the trans side, the striping of the condensed ions on the chain when passing through the pore, the counterion current that bombarded the cis-side chain near the pore were not considered or modeled in the scaling theory. These effects could play certain roles in the deviation of the results. It deserves a deeper and detailed investigation in the future. At the current stage, we can simply regard $\nu_{s}$ as a fitting parameter, which resulted, more or less, from the tension propagation picture in combination with the other omitted or unknown effects. Nevertheless, the variational behaviors of the translocation time versus *N* and *E* were generally described by the scaling theory in a good sense.

**Figure 13:**
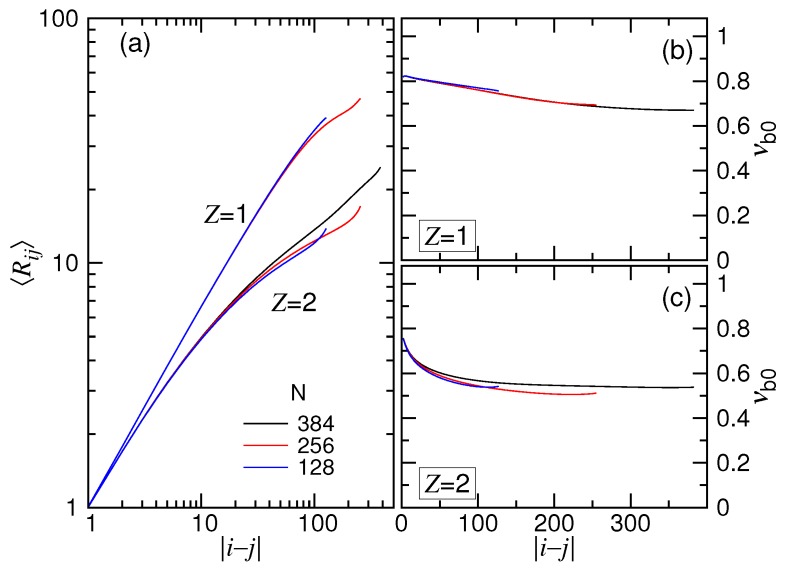
(**a**) Mean distance $\langle R_{ij}\rangle$ of a monomer pair (i,j) in the Z=1 and the Z=2 solutions. The calculated exponent $\nu_{b0}$ is plotted as a function of $\left|i-j\right|$ in (**b**) the monovalent and (**c**) the divalent solutions.

The size scaling of chain blobs was studied by the simulations using charged chains in free solutions. The exponent $\nu_{b0}$ depicts the scaling of the mean distance of a pair of monomers *i* and *j* via the relation $\langle R_{ij}\rangle ∼{\left|i-j\right|}^{\nu_{b0}}$ where $\left|i-j\right|$ is the number of the bonds that connect the two monomers. Again, the notation $\nu_{b0}$ is used, rather than $\nu_{b}$, to emphasize that the exponent was obtained under the static condition. The log-log plot of $\langle R_{ij}\rangle$ vs. $\left|i-j\right|$ is presented in Figure 13a. The exponent $\nu_{b0}$ calculated by $\log\left(\langle R_{ij}\rangle /\langle R_{i_{0}j_{0}}\rangle \right)/\log\left|i-j\right|$ are presented in Panels (b) and (c) of the figure for the Z=1 and Z=2 salt cases, respectively, where $\left|i_{0}-j_{0}\right|=1$.

Because the ionic strength is stronger, the chain acquired a smaller $\langle R_{ij}\rangle$ for the Z=2 case. The trend looks quite similar to the plot of $\langle R_{m}\rangle$ against *m*. The calculated $\nu_{b0}$ showed a decreasing behavior with $\left|i-j\right|$, similar to $\nu_{s0}$ vs. *m* as well. The difference between $\nu_{s0}$ and $\nu_{b0}$ was found not to be very important.

In Section 4.1 and Section 4.2, we have obtained q=0.86 and 0.88 from the translocation simulations in the two salt solutions. The exponent *q* was argued to relate $\nu_{b}$ to the equation $q=\left(1-\nu_{b}\right)/\left(1+\nu_{b}\right)$ by the theory. To verify this, we used the asymptotic $\nu_{b0}$ value, 0.68 for Z=1 and 0.55 for Z=2, and plugged it into the equation for $\nu_{b}$. The values q=0.19 and 0.29 were obtained for the two cases. A large discrepancy between the translocation result and the predicted result from the theory was found. This suggests that the relation equation was not held in the simulations. This is probably because the exponent *q* was obtained from the strongly-driven regimes. The blobs of the chain in these regimes could be accelerated by the imbalanced tensile forces. The local-balance assumption of forces exerted on the blobs is likely not suitable since the action time was very short. Therefore, we did not expect a good holding of the equation, which was derived under this quasi-equilibrium condition. Again, the exponent *q* could be simply treated as a fitting parameter to describe the scaling of translocation. The correct expression for *q* should be studied in the future using a more sophisticated picture to take into account the non-equilibrium feature of the blob dynamics.

### 4.4. Diffusion Behavior and the Subdiffusion Exponent $\gamma_{p}$

The diffusion behavior of translocation can be studied by calculating the variance of the translocation coordinate *n*, defined by $\langle \Delta n^{2}\rangle =\langle {\left(n-\langle n\rangle \right)}^{2}\rangle =\langle n^{2}\rangle -{\langle n\rangle }^{2}$. The variance is a time function, which measures the spreading of the variable n(t) at time *t* with respect to the mean value 〈n(t)〉. The type of diffusion can be determined from the scaling behavior $\langle \Delta n^{2}\rangle ∼t^{\gamma }$. For normal diffusion, the exponent γ is equal to one. The diffusion is subdiffusive if γ<1; it is superdiffusive if γ>1.

**Figure 14:**
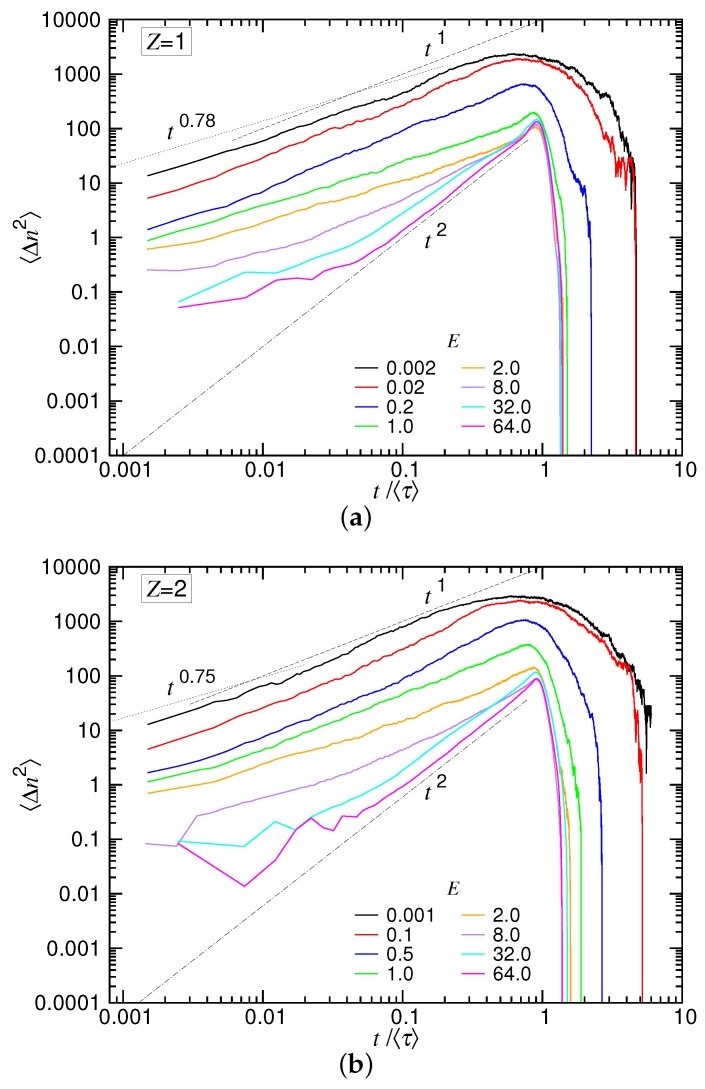
Variance of the translocation coordinate, $\langle \Delta n^{2}\rangle$, versus the normalized time, t/〈τ〉 in (**a**) the monovalent salt solution and (**b**) the divalent salt solutions. The chain has N=123. The strength of the driving field is indicated in the legend.

We calculated $\langle \Delta n^{2}\left(t\right)\rangle$ for N=123 in the monovalent and divalent salt solutions, and the results are presented in Figure 14a,b. Because the duration of translocation depends on *E*, the curves are plotted against the normalized time t/〈τ〉, rather than *t*, in order to compare them over different driving fields.

We can see that the stronger the driving field, the smaller the variance. The spreading of n(t) was thus narrowed down with increasing *E*, and the uncertainty of translocation was reduced. Departing from the weak fields, the $\langle \Delta n^{2}\rangle$ curve shows subdiffusive behavior in the small *t* region. The value of γ was found to be 0.78 and 0.75 for the two salt cases, respectively. This is the exponent $\gamma_{p}$, which describes the memory effect in the tension propagation stage. $\langle \Delta n^{2}\rangle$ showed normal diffusion behavior (γ≃1) as *t* further increased. Around t≃〈τ〉, the curve was rounded off and decreased rapidly to zero at about five-times the mean translocation time. The fact that the variance went eventually to zero is not unexpected because the translocation ended with the entire chain arriving at the trans region, and thus, the coordinate *n* was *N* and had no variation. In the strong driving situations (E≥32.0), the process started with a subdiffusive behavior and soon evolved to be a super-diffusive one as t/〈τ〉>0.1 with the exponent γ equal to two. It shows that the main course of the translocation was primarily dominated by a ballistic type of motion. Near t/〈τ〉≃1, there was a surge on the curve, which suggests an acceleration due to the entropic pulling from the trans-side chain, before the ending occurred at *t* about 1.3〈τ〉.

### 4.5. Discussion

Although the scaling theory was derived for neutral chains, the translocation behavior of charged polymers can be mainly described by it. For example, the predicted scalings in the four force regimes and the subdiffusive feature of translocation were observed in the simulations for both of the monovalent and divalent salt cases. We summarize the exponents obtained in this study in Table 1, including those directly extracted from the simulations and the ones calculated from the scaling theory.

We saw that the reported exponents for the two salt cases have no big differences. However, the dependence of the exponents on the salt valence is not trivial; some of them increase with *Z*, and the others decrease. In the table, the three α’s (denoted by $\alpha_{\mathrm{UB}}$, $\alpha_{\mathrm{SD}(T)}$ and $\alpha_{\mathrm{SD}(I)}$) obtained in the UB, SD(T) and SD(I) force regimes are given. They can be used to solve the three exponents $\nu_{s}$, *q* and $z_{p}$. In Section 4.3, it has been shown that the calculated $\nu_{s}$ value is significantly different from the static exponent $\nu_{s0}$ and also *q* deviates from the one computed using the other static exponent $\nu_{b0}$. Nevertheless, the consistency of the theory can be verified via the third exponent $z_{p}$, which is 5.06 and 4.88 in the two salt solutions. The scaling theory demands $z_{p}$ to be connected with $\nu_{s}$ and $\gamma_{p}$ by the relation $\gamma_{p}=\left(1+\nu_{s}\right)/\left(z_{p}\nu_{s}\right)$. Plugging the computed $\nu_{s}$ and the measured $\gamma_{p}$ (in Figure 14) into the relation yields $z_{p}=4.95$ and 4.00 for the two cases, which are quite in accordance with the above results.

The dynamics of tension propagation for a pulled linear chain in free solutions has been investigated by Rowghanian and Grosberg [89]. The tension was found to propagate in the way $m_{p}∼t^{1/2}$, which gives the dynamical exponent $z_{p}=2/\nu_{s0}$ (in our notations). The predicted $z_{p}$, according to their theory, is 2.82 and 3.51, respectively, using the $\nu_{s0}$ value of our simulations. The exponent is found smaller than ours. The difference simply comes from different problems treated. In a translocation problem, a tension blob must be destroyed before threading it into the pore, which did not occur in the pulling-chain problem in free solutions. The distribution of the tension blobs, and thus the propagation of tension, on the cis side is dynamically regulated in some way; it renders the problem more complicated. The details of the scaling of tension propagation in polymer and polyelectrolyte translocation will be investigated in the future.

The exponents ρ and η were introduced in Section 2 to define the scaling boundaries of the force regimes. To maintain the sense of the scaling theory and the four predicted force regimes, certain inequalities given in the section should be held; they are $\nu_{s}z_{p}\gamma_{p}<2$, $\nu_{s}\rho <1$, ρ>η, $p_{z}\left(1-q\right)>1$ and $z_{p}\left(1-\gamma_{p}\right)>1$. One can easily verify that the reported exponents satisfy all these inequalities.

**Figure 15:**
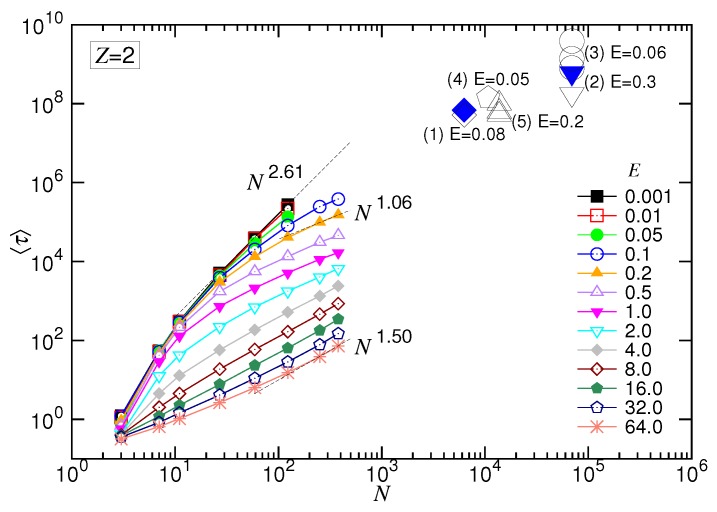
Comparison of the experimental results with the simulations. The experimental data are taken from (1) [85] (diamonds), (2) [86] (inverted triangles), (3) [82] (circles), (4) [105] (pentagons) and (5) [106] (triangles). The big blue solid symbols indicate that the translocation was done in the presence of divalent salt, while the big open symbols indicate that the data were acquired in the monovalent salt solutions. The strength of the transmembrane field is given near the data points. The conversion of the experimental data into our simulation units can be found in the Supplemental Materials.

Experiments have reported that the duration time can be significantly increased if DNA translocation is performed in the presence of divalent salts [85,86]. Uplinger et al. [85] studied the translocation of a circular supercoiled DNA (pBR322) of 4.4kbp in a 1.6M KCl solution. They found that adding MgCl${}_{2}$ salt of concentration 100mM into the solution slowed down the translocation time from 110μs to 145μs when the system was biased by a transmembrane electric field E=120mV/15nm=8mV/nm. Since the length unit σ in our simulations is about 0.7bp, the time unit $t_{u}≃2.13\;\mathrm{ps}$ and the electric field strength unit $E_{u}≃100\;\mathrm{mV}/\mathrm{nm}$ (see the Supplemental Materials and [72] for the explanation), the experiment has the corresponding chain length N=6286, the translocation time $\tau =6.8\times 10^{7}$ and the driving field E=0.08. Zhang et al. [86] studied linear λ-DNA of 48.8kbp threading through a nanopore. They reported that the mean translocation time increased about three-fold, from 0.38ms to 1.31ms, in the driving field E=600mV/20nm=30mV/nm, if the 1M KCl solution was replaced by the 1M MgCl${}_{2}$ one. Converting their data with the simulation units gives N= 69,286, $\tau =6.2\times 10^{8}$ and E=0.3. We plot the two experimental data (in big blue solid symbols), τ versus *N*, for the divalent salt case in Figure 15, regardless of the differences of the systems between the driving field, salt concentration, ion size, chain structure, chain stiffness, pore diameter, membrane thickness, and so on.

Impressively, the data are very compatible with the trend of simulations, although the simulations were done by a simple model of charged bead-spring chains, where many complex issues, such as the charged wall surface, the electroosmotic flow, the formation of a funnel-shaped electric field potential near the pore entrance, were not considered and the chains were not very long. The experimental data obtained in monovalent salt solutions in [82,85,86,105,106] have also been converted and plotted, in addition, on the figure (in big open symbols) for comparison. The differences between the two sets of translocation time are not big in the scaling (log-log) plot, since the reported gain for a replacement of the monovalent salt by the divalent one is of only a few folds. Therefore, the exponent α in the divalent salt solutions would not be significantly larger than the one in the monovalent salt solutions. It is in accordance with what we found in the simulations.

In view of the locations of the data on the figure, we know that the experiments were probably studied in the weakly-driven (WD) situation. It essentially followed the demand of applications, to slow down the threading process using a weak field, in order to gain better resolution in the detection. To investigate translocation behavior in the SD(T) or SD(I) force regime, either the strength of the driving field should be increased or a much longer chain should be used in experiments, according to the scaling picture depicted in Figure 2b.

The upper bound of translocation time was obtained in this study because the chain head was initially placed across the pore and interdicted, by assumption, from reentering the pore. As a result, failure by retracting the entire chain into the cis side will not occur. Thus, even in a zero driving field, the translocation is still possible, which is realized by a random walk to overcome the entropic barrier created by the chain body on the cis side. In usual experiments of DNA translocation, there is no such mechanism to prohibit the reentering of the head monomer into the pore. Therefore, the translocation can fail, and the probability of failure increases rapidly as the bias field is lowered. To investigate experimentally the upper bound, end-labeling a DNA molecule with a bead larger than the pore size might be a possible way to give the required mechanism.

## 5. Conclusions

In this work, we have rederived the scaling theory for polymer translocation. Four force regimes, namely the unbiased (UB) regime, the weakly-driven (WD) regime, the strongly-driven regime with the trumpet-shaped ensemble of tension blobs (SD(T)) and the strongly-driven regime under the isoflux condition (SD(I)), were distinguished and analyzed. The mean translocation time has been shown to scale as $\langle \tau \rangle ∼N^{\alpha }f^{-\delta }$, with the pair of the exponents (α,δ) being $\left(2+\nu_{s}z_{p}\left(1-\gamma_{p}\right),0\right)$, $\left(1+\nu_{s}z_{p}\left(1-\gamma_{p}\right),1\right)$, $\left(1+\nu_{s}\left(1-q\right)\right),p_{z}\left(1-q\right))$ and (1+ν,1) in the four force regimes, respectively. To verify the theory, Langevin dynamics simulations have been performed by using charged polymers with explicit ions in the study. Two kinds of adding salts, the monovalent salt and the divalent salt, were considered. The strength of the driving electric field *E* was varied over five orders of magnitude to explore the full force regimes. The obtained 〈τ〉 vs. *E* curves and the 〈τ〉 vs. *N* curves showed the characteristic scaling behaviors depicted by the theory well, although the theory was developed under the framework of neutral polymers. The key exponents $\nu_{s}$, *q* and $z_{p}$ were extracted directly from the simulations; the discrepancy between these exponents and those calculated from the static exponents $\nu_{s0}$ and $\nu_{b0}$ has been pointed out. The subdiffusion exponent $\gamma_{p}$ has been studied as well, from the calculation of the variance of the translocation coordinate. The variations of the scalings with the salt valence *Z* can be found in Table 1. Furthermore, the obtained exponents were verified to satisfy a bunch of required inequalities. It reinforces the validness of the scaling theory and the depicted physical pictures. Finally, the simulation results were compared with the experimental data. Good compatibility was found, and the possible location of the experiments on the scaling plot has been identified.

## Figures and Tables

**Table 1:** List of the exponents in the (*Z*:1)-salt solution obtained in this study. Some of the exponents were extracted directly from the simulations. For these, the figure number from which the exponent was obtained is given (in the note column). For the exponents calculated indirectly from the extracted exponents, the word “calc.” is denoted, following the item numbers from which it was calculated.

Item No.	Exponent	Z=1	Z=2	Note
1	$\alpha_{\mathrm{UB}}=2+\nu_{s}z_{p}\left(1-\gamma_{p}\right)$	2.39	2.61	Figure 5 & Figure 9
2	$\alpha_{\mathrm{SD}(T)}=1+\nu_{s}\left(1-q\right)$	1.05	1.06	Figure 5 & Figure 9
3	$\alpha_{\mathrm{SD}(I)}=1+\nu_{s}$	1.35	1.50	Figure 5 & Figure 9
4	$\delta_{\mathrm{SD}(T)}=p_{z}\left(1-q\right)$	1.65	1.77	Figure 4 & Figure 8
5	$\nu_{s0}$	0.71	0.57	Figure 12
6	$\nu_{b0}$	0.68	0.55	Figure 13
7	$\gamma_{p}$	0.78	0.75	Figure 14
8	$\nu_{s}$	0.35	0.50	calc.; item 3
9	*q*	0.86	0.88	calc.; items 2, 8
10	$z_{p}$	5.06	4.88	calc.; items 1, 7, 8
11	$\rho =\frac{z_{p}\left(1-\gamma_{p}\right)-1+q}{p_{z}\left(1-q\right)-1}$	1.50	1.43	calc.; items 4, 9, 10
12	$\eta =\frac{q}{p_{z}\left(1-q\right)-1}$	1.32	1.14	calc.; items 4, 9

## Supplementary Materials

The following is available online at http://www.mdpi.com/2073-4360/10/11/1229/s1: 1. Converting the experimental data into the simulation units.

## Appendix A

Consider a one-dimensional generalized Langevin equation (1D GLE):

(A1) $$m\ddot{x}+\int_{0}^{t}K\left(t-t^{\prime }\right)\dot{x}\left(t^{\prime }\right)dt^{\prime }=-\frac{d\;U(x)}{d\;x}+\xi \left(t\right)$$

where K(t) is the memory kernel and ξ(t) is the noise with zero mean. The generalized fluctuation-dissipation theorem states $\langle \xi \left(t\right)\xi \left(t^{\prime }\right)\rangle =k_{B}T\;K(|t-t^{\prime }\left|\right)$ [107]. For a power-law decay memory kernel $K\left(t\right)=\frac{k_{0}}{\Gamma (1-\gamma )}t^{-\gamma }$ where $k_{0}$ is a constant and Γ(x) is the gamma function, the velocity autocorrelation function $C_{\dot{x}\dot{x}}$ and the mean square displacement of *x* can be shown to be $C_{\dot{x}\dot{x}}\left(t\right)≃\frac{k_{B}T}{k_{0}}\frac{t^{\gamma -2}}{\Gamma (\gamma -1)}$ and $\langle \Delta x{\left(t\right)}^{2}\rangle ≃\frac{2k_{B}T}{k_{0}}\frac{t^{\gamma }}{\Gamma (\gamma +1)}$, respectively [108].

We adopted the results for a translocation in the UB regime and phenomenologically assumed that the variance of the translocation coordinate scales as $\langle \Delta n{\left(t\right)}^{2}\rangle ∼t^{\gamma_{p}}$, which is originated from a memory kernel of the form $K\left(t\right)∼t^{-\gamma_{p}}$. According to Panja et al. and Sakaue [15,25], the kernel can be estimated from the relation $K\left(t\right)\Delta m_{p}∼f\left(t\right)$ where the tension force f(t) has a magnitude computed from the entropic force of the chain by:

(A2) $$f\left(t\right)∼\frac{k_{B}T}{R_{p}^{2}}\Delta R_{p}∼k_{B}T\nu_{s}m_{p}^{-1-\nu_{s}}\Delta m_{p}∼t^{-\left(1+\nu_{s}\right)/\left(\nu_{s}z_{p}\right)}\Delta m_{p}$$

Here, the scaling relations $R_{p}∼m_{p}^{\nu_{s}}$ and $R_{p}∼t^{1/z_{p}}$ have been used in the derivation. The obtained kernel thus has the exponent $\gamma_{p}$ equal to $\left(1+\nu_{s}\right)/\left(\nu_{s}z_{p}\right)$.
