# Supplementary material for: Translocation of Charged Polymers through a Nanopore in Monovalent and Divalent Salt Solutions: A Scaling Study Exploring over the Entire Driving Force Regimes
Source: Polymers (Basel). 2018 Nov 6;10(11):1229. doi: 10.3390/polym10111229 (PMC6290626; doi:10.3390/polym10111229)
Supplement: Supplementary file 1 [file polymers-10-01229-s001.pdf]

Article

Supplementary Materials: Translocation of Charged Polymers through a Nanopore in Monovalent and Divalent Salt Solutions: A Scaling Study Exploring over the Entire Driving Force Regimes

Pai-Yi Hsiao [1,2*]

[1] Department of Engineering and System Science, National Tsing Hua University, Hsinchu, Taiwan, ROC
[2] Institute of Nuclear Engineering and Science, National Tsing Hua University, Hsinchu, Taiwan, ROC
* Correspondence: pyhsiao@ess.nthu.edu.tw; Tel.: +886-3-516-2247

Academic Editor: name
Version October 31, 2018 submitted to Polymers; Typeset by LaTeX using class file mdpi.cls

---

1. Converting the experimental data into the simulation units

In our simulations, the length unit, time unit, and electric field strength unit are, respectively,

$$\begin{aligned} \sigma &= \lambda_{\mathrm{B}}/3.0 \simeq 2.38 \times 10^{-10}\,\mathrm{m} \simeq 0.7\,\mathrm{bp} \\ t_{\mathrm{u}} &= \sigma\sqrt{m/k_{\mathrm{B}}T} \simeq 2.13 \times 10^{-12}\,\mathrm{s} \\ E_{\mathrm{u}} &= k_{\mathrm{B}}T/(e\sigma) \simeq 100\,\mathrm{mV/nm} \end{aligned}$$

The explanation, how to obtain these values, can be found in Ref. [1].

Followings are the translocation time reported in five experimental papers. The data converted with our simulation units are given behind the symbol ' $\Longrightarrow$ '.

(1) Experiment by Uplinger et al. [2]:

- molecules: circular supercoiled DNA (pBR322) of ~4.4 kbp $\Longrightarrow N = 6286$
- pore diameter: ~14 nm
- transmembrane field: $E = 120\,\mathrm{mV}/15\,\mathrm{nm} = 8\,\mathrm{mV/nm}$ $\Longrightarrow E = 0.08$
- translocation time:
  - (i) $\tau \simeq 110\,\mu\mathrm{s}$ in 1.6 M KCl solution $\Longrightarrow \tau = 5.16 \times 10^7$
  - (ii) $\tau \simeq 145\,\mu\mathrm{s}$ in 1.6 M KCl + 100 mM $MgCl_2$ solution $\Longrightarrow \tau = 6.81 \times 10^7$

(2) Experiment by Zhang et al. [3]:

- molecules: λ-dsDNA, linear, ~48.5 kbp long $\Longrightarrow N = 69286$
- pore diameter: ~20 nm
- transmembrane field: $E = 600\,\mathrm{mV}/20\,\mathrm{nm} = 30\,\mathrm{mV/nm}$ $\Longrightarrow E = 0.3$
- translocation time:
  - (i) $\tau \simeq 0.38$ ms in 1 M KCl solution $\Longrightarrow \tau = 1.78 \times 10^8$
  - (ii) $\tau \simeq 1.31$ ms in 1 M $MgCl_2$ solution $\Longrightarrow \tau = 6.15 \times 10^8$

(3) Experiment by Kowalczyk et al. [4]:

- molecules: λ-dsDNA, linear, ~48.5 kbp long $\Longrightarrow N = 69286$
- pore diameter: ~15.3 nm
- transmembrane field: $E = 120\,\mathrm{mV}/20\,\mathrm{nm} = 6\,\mathrm{mV/nm}$ $\Longrightarrow E = 0.06$
- translocation time:
  - (i) $\tau \simeq 1.72$ ms in 1 M KCl solution $\Longrightarrow \tau = 8.08 \times 10^8$
  - (ii) $\tau \simeq 2.94$ ms in 1 M NaCl solution $\Longrightarrow \tau = 1.38 \times 10^9$

(iii) $\tau \simeq 8.23\,\mathrm{ms}$ in 1 M LiCl solution $\Longrightarrow \tau = 3.86 \times 10^9$

(4) Experiment by Krueger et al. [5]:

- DNA molecules: circular plasmid pTYB21, 7514 bp long $\Longrightarrow N = 10734$
- pore diameter: ~20 nm
- transmembrane field: $E = 100\,\mathrm{mV}/\,20\,\mathrm{nm} = 5\,\mathrm{mV/nm}$ $\Longrightarrow E = 0.05$
- translocation time: $\tau \simeq 300\,\mu\mathrm{s}$ in 1 M KCl solution $\Longrightarrow \tau = 1.41 \times 10^8$

(5) Experiment by Ito et al. [6]:

- molecules: 9.6 kbp DNA, linear $\Longrightarrow N = 13714$
- pore diameter: $10 - 25\,\mathrm{nm}$
- transmembrane field: $E = 300\,\mathrm{mV}/\,15\,\mathrm{nm} = 20\,\mathrm{mV/nm}$ $\Longrightarrow E = 0.2$
- translocation time:

(i) $\tau \simeq 110\,\mu\mathrm{s}$ in 1 M KCl solution $\Longrightarrow \tau = 5.16 \times 10^7$
(ii) $\tau \simeq 130\,\mu\mathrm{s}$ in 1 M NaCl solution $\Longrightarrow \tau = 6.10 \times 10^7$
(iii) $\tau \simeq 210\,\mu\mathrm{s}$ in 1 M LiCl solution $\Longrightarrow \tau = 9.86 \times 10^7$

References

1. Hsiao, P.Y. Conformation Change, Tension Propagation and Drift-Diffusion Properties of Polyelectrolyte in Nanopore Translocation. *Polymers* **2016**, *8*, 378.
2. Uplinger, J.; Thomas, B.; Rollings, R.; Fologea, D.; McNabb, D.; Li, J. K^+, Na^+, and Mg^{2+}on DNA translocation in silicon nitride nanopores. *Electrophoresis* **2012**, *33*, 3448–3457.
3. Zhang, Y.; Liu, L.; Sha, J.; Ni, Z.; Yi, H.; Chen, Y. Nanopore detection of DNA molecules in magnesium chloride solutions. *Nanoscale Res. Lett.* **2013**, *8*, 245.
4. Kowalczyk, S.W.; Wells, D.B.; Aksimentiev, A.; Dekker, C. Slowing down DNA Translocation through a Nanopore in Lithium Chloride. *Nano Lett.* **2012**, *12*, 1038–1044.
5. Krueger, E.; Shim, J.; Fathizadeh, A.; Chang, A.N.; Subei, B.; Yocham, K.M.; Davis, P.H.; Graugnard, E.; Khalili-Araghi, F.; Bashir, R.; Estrada, D.; Fologea, D. Modeling and Analysis of Intercalant Effects on Circular DNA Conformation. *ACS Nano* **2016**, *10*, 8910–8917.
6. Ito, S.; Yamazaki, H.; Tsukahara, M.; Esashika, K.; Saiki, T. Salt dependence of DNA translocation dynamics through silicon nanopores detected by ultraviolet excitation. *Appl. Phys. A* **2016**, *122*.

© 2018 by the authors. Submitted to *Polymers* for possible open access publication under the terms and conditions of the Creative Commons Attribution license (http://creativecommons.org/licenses/by/4.0/)
